# LibKiSAO: a Java library for Querying KiSAO

**DOI:** 10.1186/1756-0500-5-520

**Published:** 2012-09-24

**Authors:** Anna Zhukova, Richard Adams, Camille Laibe, Nicolas Le Novère

**Affiliations:** 1 EMBL-EBI, Wellcome Trust Genome Campus, Hinxton, CB10 1SD, United Kingdom; 2 SynthSys Edinburgh, University of Edinburgh, Edinburgh EH9 3JD, United Kingdom; 3 Babraham Institute, Babraham Research Campus, Cambridge, CB22 3AT, United Kingdom

**Keywords:** Java library, Simulation, Algorithm search, Ontology

## Abstract

**Background:**

The Kinetic Simulation Algorithm Ontology (KiSAO) supplies information about existing algorithms available for the simulation of Systems Biology models, their characteristics, parameters and inter-relationships. KiSAO enables the unambiguous identification of algorithms from simulation descriptions. Information about analogous methods having similar characteristics and about algorithm parameters incorporated into KiSAO is desirable for simulation tools. To retrieve this information programmatically an application programming interface (API) for KiSAO is needed.

**Findings:**

We developed libKiSAO, a Java library to enable querying of the KiSA Ontology. It implements methods to retrieve information about simulation algorithms stored in KiSAO, their characteristics and parameters, and methods to query the algorithm hierarchy and search for similar algorithms providing comparable results for the same simulation set-up. Using libKiSAO, simulation tools can make logical inferences based on this knowledge and choose the most appropriate algorithm to perform a simulation. LibKiSAO also enables simulation tools to handle a wider range of simulation descriptions by determining which of the available methods are similar and can be used instead of the one indicated in the simulation description if that one is not implemented.

**Conclusions:**

LibKiSAO enables Java applications to easily access information about simulation algorithms, their characteristics and parameters stored in the OWL-encoded Kinetic Simulation Algorithm Ontology. LibKiSAO can be used by simulation description editors and simulation tools to improve reproducibility of computational simulation tasks and facilitate model re-use.

## Findings

### Background

**Figure 1:**
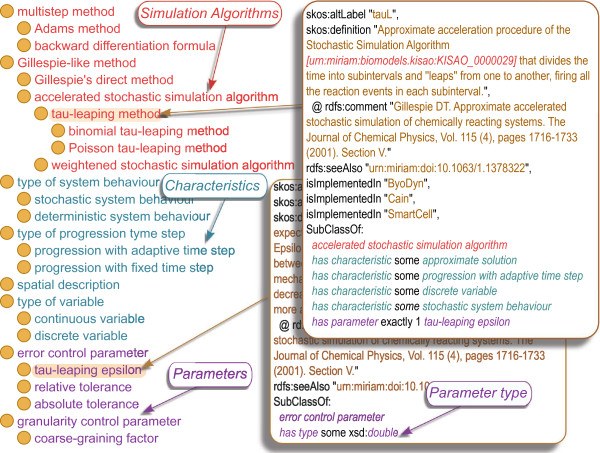
**Structure of KiSAO.** The Kinetic Simulation Algorithm Ontology (KiSAO) consists of three main branches representing the hierarchy of simulation algorithms, their characteristics and their parameters. The relationships *has characteristic* and *has parameter* are used to link algorithms to the characteristics they possess and parameters they use. The algorithms stored in KiSAO are annotated with their names, synonymous names, descriptions and links to the articles describing them. Information about parameter types is also incorporated into KiSAO.

The reproducibility of a computational simulation task is of crucial importance in systems biology, and requires that both the algorithm used and the initial set-up need to be identified. These minimum information requirements are described by the MIASE guidelines [1]. The Kinetic Simulation Algorithm Ontology (KiSAO) [2] addresses the issue by classifying algorithms available for the simulation of Systems Biology models (e.g. *tau-leaping method*, *finite element method*) which allows them to be identified unambiguously from simulation descriptions, such as SED-ML [3]. Information about algorithm characteristics (e.g. *progression with adaptive time steps*, *stochastic system behaviour*) incorporated into KiSAO enables the identification of similar algorithms. This knowledge helps simulation software to automatically choose the best algorithm available to perform a simulation, and, if the original one is not implemented, to use an alternative algorithm that will provide comparable results. The structure of KiSAO is represented in Figure 1.

Since KiSAO is implemented in OWL 2 [4], developers of libraries and tools processing simulation description languages could potentially work with KiSAO using general programming interfaces for manipulating ontologies, such as OWL API [5], and ontology reasoners, such as HermiT [6]. Richer programming interfaces for interacting with ontology resources are also available, e.g., OntoCAT [7], an application suite enabling the querying of several resources simultaneously, looking for synonyms and definitions (which is not supported directly by OWL API). Nevertheless, due to the complex structure and variety of relationships used in KiSAO, none of these interfaces provide an easy way to retrieve all the information from KiSAO. Moreover, some operations require the knowledge of how KiSAO is organised. For instance, to find similar simulation algorithms both the hierarchical structure of the algorithm sub-tree of KiSAO, and algorithm-characteristic relationships should be taken into account. It is more efficient and convenient for developers to use a higher-level, KiSAO-specific API. We therefore developed libKiSAO to address this need.

### Implementation

LibKiSAO is a Java library which uses the OWL API [5] and the HermiT reasoner [6] to implement an intuitive interface for querying KiSAO. It is available under the LGPL license for anyone to use and extend. We chose the Java language for the implementation because of its platform independence and wide adoption. The compatibility requirements of the Hermit reasoner restricted us to Java version 1.5 or higher.

LIbKiSAO consists of two parts. The first part is a general purpose ontology querying library libOnto, wrapping some of the OWL API low-level functionality to implement shorter methods, which are more clear for non-ontology specialists. Examles are *getSynonyms()*, *getPropertyValues()*, *searchByName()*, which are not implemented natively in the OWL API but are often used while working with ontologies. LibOnto can be used separately from libKiSAO for various ontology-querying purposes and is independent of any knowledge about the KiSA Ontology. An Ant build script which packs libOnto into a separate jar archive is available for download from the project website [8].

The second part of libKiSAO is represented by the interface *http:// net.biomodels.kisao.IKiSAOQueryMaker* and its implementation *net.biomodels.kisao.impl.KiSAOQueryMaker*. It implements KiSAO-specific functionality and uses libOnto to work with OWL. The implemented KiSAO-querying methods are described in detail in Tables 1, 2. LibKiSAO allows users to choose any particular version of KiSAO they are interested in working with, stored as a local file or on the web, by specifying a constructor parameter. By default the latest release of KiSAO (referred to by the KiSAO URI [9]) is selected.

**Table 1:** Taxonomy level querying methods implemented in libKiSAO

**Method name**	**Description**
** Listing**	
getAllAlgorithms()	Lists simulation algorithms stored in KiSAO
getAllCharacteristics()	Lists simulation algorithm characteristics stored in KiSAO
getAllParameters()	Lists simulation algorithm parameters stored in KiSAO
getType(entry)	Returns type (algorithm, characteristic or parameter) of the
	specified entry
isAlgorithm(entry)	Checks if the specified entry represents simulation algorithm
	in KiSAO
isCharacteristic(entry)	Checks if the specified entry represents simulation algorithm
	characteristic in KiSAO
isParameter(entry)	Checks if the specified entry represents simulation algorithm
	parameter in KiSAO
** Search**	
searchById(id)	Looks for the entry with the specified identifier or miriam urn
searchByName(name)	Looks for the entries with the specified name or synonym
** Retrieving Element Information**	
getName(entry)	Returns name of the specified entry
getAllSynonyms(entry)	Returns synonyms of the specified entry
getSynonyms(entry, type)	Returns synonyms of the specified type (exact, related,
	broad or narrow)
getDefinition(entry)	Returns definition of the specified entry
getLinks(entry)	Returns links to articles/books that describe the specified entry
getId(iri)	Returns id (e.g. kisao:0000001) of the specified entry
getMiriamURN(entry)	Returns MIRIAM URN (e.g. urn: miriam: biomodels.kisao:
	KISAO_0000001) of the specified entry
getIdentifiersOrgURL(entry)	Returns identifiers.org URL of the specified entry
isDeprecated(entry)	Checks whether the specified entry is deprecated
** Querying Hierarchy**	
getAncestors(entry, direct)	Returns ancestors of the specified entry
getDescendants(entry, direct)	Returns descendants of the specified entry
isA(descendantCandidate, ancestorCandidate)	Checks if a descendantCandidate entry is a descendant
	(concerning rdf:subClassOf relationship) of the
	ancestorCandidate entry in KiSAO

The table lists the methods implemented in libKiSAO which enable querying the taxonomy level of KiSAO.

**Table 2:** Complex/application level querying methods implemented in libKiSAO

**Method name**	**Description**
** Querying for Algorithm Characteristics**	
getCharacteristics(algorithm, *optional* type(s))	Returns characteristics of the specified algorithm. An optional
	type(s) parameter filters the returned characteristic set by
	their type(s)
hasCharacteristic(algorithm, characteristic(s))	Checks if the specified algorithm possesses the specified
	characteristic(s)
** Looking for Similar Algorithms**	
getAlgorithmsByCharacteristic	
(characteristic(s))	Returns algorithms which possess specified characteristic(s)
getAlgorithmsWithSameCharacteristics	
(algorithm, *optional* type(s))	Returns algorithms having the same characteristics as the
	specified one. An optional type(s) parameter specifies which
	types of characteristics should be considered. By default,
	all the characteristics are considered
getNMostSimilarAlgorithms(algorithm, n,	
*optional* type(s))	Returns n algorithms which are most similar to the specified
	one, sorted by the distance in the hierarchy tree. An
	optional type(s) parameter specifies which types of
	characteristics should be considered when looking for
	similar algorithms. By default, all the characteristics
	are considered
getAlgorithmsByQuery(query)	Returns algorithms described by the specified query
** Querying for Algorithm Parameters**	
getParameters(algorithm)	Returns parameters that are used by the specified algorithm
getParametersByCharacteristic(characteristic(s))	Returns parameters that are used by the algorithms with the
	specified characteristic(s)
hasParameter(characteristics, parameter(s))	Checks if the algorithm described by its characteristics uses
	the specified parameter(s)
getParametersByAncestorAndCharacteristic	
(ancestor, characteristic(s))	Returns a set of parameters that are used by the algorithms
	with the specified characteristic(s) and ancestor
hasParameter(algorithm, parameter(s))	Checks if the specified algorithm uses the specified
	parameter(s)
getParameterType(parameter)	Returns the type of the specified parameter
** Querying for Inter-algorithm Relationships**	
isHybrid(algorithm)	Checks whether the specified algorithm is a hybrid one
getHybridOf(algorithm)	Returns collection of the algorithms the specified one is a
	hybrid of

The table lists the methods implemented in libKiSAO which enable querying the complex/application level of KiSAO.

KiSAO-specific library part implements such methods as looking for algorithms having particular characteristics or algorithm grouping methods with similarity distance based on algorithm characteristics and hierarchy relationships.

A user can specify which characteristics should be considered while looking for similar methods. For example, *getNMostSimilarAlgorithms(poissonTauLIri, 4, KiSAOIRI.TYPE_OF_SYSTEM_BEHAVIOUR_IRI, KiSAOIRI.TYPE_OF_VARIABLE_IRI)* returns 4 algorithms having the same type of system behaviour (stochastic) and of variables (discrete) as the Poisson tau-leaping method, and sorted by the hierarchical distance to it: tau-leaping method, multinomial tau-leaping method, binomial tau-leaping method, and implicit tau-leaping method. Other characteristics are not taken into account (e.g. method type can be implicit as well as explicit).

If the distance in the hierarchy tree is not important, the method *getAlgorithmsWithSameCharacteristics(IRI.. characteristicType)* can be used. By default (if no characteristic types are specified) all the characteristics are looked at.

Currently all the characteristics are equally important for the algorithms finding similar simulation methods. In the future they might be prioritized to improve the similarity measurement.

Code examples and Java documentation are available both for download and on-line from the project website [8].

### Results and discussion

LibKiSAO supports working with KiSAO at taxonomic and complex/application levels [10]. The taxonomic layer represents a hierarchy of simulation algorithms, annotated with the information about their names, definitions, and references to the articles they are described in. For instance, as shown in Figure 1, the *tau-leaping method* is an ancestor of the *binomial tau-leaping method* and is annotated with the synonym *tauL*. The complex and application levels of KiSAO enable algorithm grouping based on characteristics (e.g. *stochastic*/*deterministic system behaviour*), link algorithms to the parameters they use (e.g. *tau-leaping method’has parameter’tau-leaping epsilon*), and describe parameter types (e.g. *tau-leaping epsilon’has type’xsd:double*).

LibKiSAO provides Java applications with the mechanisms to retrieve this information programmatically from the OWL-encoded KiSAO. LibKiSAO can be used by simulation description language manipulating libraries, such as jlibSEDML [11], and simulation tools, such as COPASI [12].

#### Taxonomy level querying methods

LibKiSAO provides methods for listing simulation algorithms stored in KiSAO and searching for them by names, synonymous names, MIRIAM URIs [13] and identifiers. Retrieving algorithm descriptions, synonyms and references to the articles describing the algorithm is also implemented, as well as methods for querying algorithm hierarchy, such as searching for descendant (derived from, more specific) and ancestor (more general) algorithms. The supported taxonomy querying methods are listed in Table 1. Use case 1 describes how querying KiSAO taxonomy level capabilities of libKiSAO can be used by simulation description language editors, such as SED-ED [14], a tool for editing, viewing and running SED-ML files.

##### Use Case 1: Using libKiSAO for querying the taxonomy level of KiSAO

**Figure 2:**
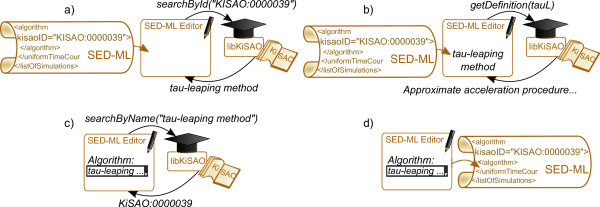
**Use of libKiSAO by simulation description editors.** LibKiSAO provides simulation description editing tools with a mapping between KiSAO identifiers used in SED-ML and human-readable information about the algorithms.

A simulation experiment encoded in SED-ML includes the information about the algorithm(s) to use. The algorithm is referenced by its KiSAO identifier, e.g. *<algorithm kisaoID=”KiSAO:0000088”>*. Since this alphanumeric identifier does not provide human-readable information, the SED-ML editing tool should also present the algorithm name and its description in the graphical user interface. Using libKiSAO, SED-ML editors can easily map algorithm identifiers used in SED-ML to the human-readable information describing them. Figures 2a,b show such a mapping for the *tau-leaping method*: The SED-ML file contains the algorithm identifier (*’KISAO:0000039’*), which can be parsed by libraries, such as jlibSEDML, and then, using libKiSAO, mapped to the algorithm name, description and other information about the algorithm contained in KiSAO. This information can then be displayed to the user. The reverse mapping is also possible: Having an algorithm chosen in the user interface, a simulation description editor can easily obtain its KiSAO identifier using libKiSAO, as shown in Figures 2c,d.

#### Complex/application level querying capabilities

LibKiSAO implements characteristic-based grouping of the simulation algorithms, stored in KiSAO, by providing methods for searching for the algorithms which possess/lack given characteristics, as well as querying for the characteristics of a given algorithm. LibKiSAO also includes methods for retrieving information about simulation algorithm parameters and their types. The complex/application level querying methods available are described in more details in Table 2. These features enable simulation tools dealing with SED-ML descriptions to automatically handle a wider range of simulation tasks, as it is discussed in use case 2.

##### Use Case 2: Using libKiSAO for querying the complex/application level of KiSAO

**Figure 3:**
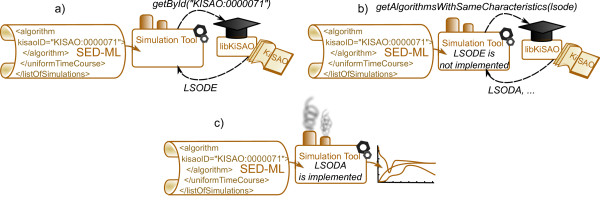
**Use of libKiSAO by simulation tools.** LibKiSAO provides simulation tools with information about available simulation methods, as well as analogous methods which can be used if the one indicated in the SED-ML description is not implemented.

Simulation tools use simulation descriptions as an input and provide numerical results as an output. Given a simulation description containing a KiSAO identifier of a simulation algorithm (e.g. *KiSAO:00000071*), a simulation tool first needs to identify the algorithm (e.g. *LSODE*). This can be done using libKiSAO (see Figure 3a). Since a simulation tool is unlikely to implement all the algorithms available for simulation of Systems Biology models, it is possible that the method indicated in the simulation description is not available (Figure 3b). The knowledge of algorithms having similar characteristics incorporated into KiSAO, and accessible via libKiSAO, will help simulation tools to choose an alternative method having similar characteristics, among those that they implement (Figure 3c).

In the future versions of SED-ML not only defining an identifier of a simulation algorithm, but also its parameters and their values might be supported [15]. In this case libKiSAO methods for extracting KiSAO knowledge about parameters common for different algorithms, will help simulation tools to switch between similar algorithms easier.

LibKiSAO therefore provides simulation tools with additional knowledge about available simulation methods, allowing them to automatically handle even the simulation descriptions referring to algorithms they cannot access.

#### Future work

The ability to choose related algorithms is also desirable in a web-service based environment. Since a particular solver service may not always be available, it is important to determine ones which can be used instead. LibKiSAO functionality to indicate similar algorithms can help to search for alternatives from a list of simulation providers, such as Biocatalogue [16], the life science web services registry.

### Conclusions

We have developed a Java library for querying the Kinetic Simulation Algorithm Ontology. LibKiSAO helps modellers to encode their simulation tasks in unambiguous languages, such as SED-ML, which can be understood by other modellers and also by simulation tools. It enables accurate and repeatable execution of computational simulation tasks and facilitates the re-usability of models in Systems Biology.

## Availability and requirements

**Project name:** libKiSAO**Project home page:**http://biomodels.net/kisao/libkisao.html**Operating system(s):** Platform independent**Programming language:** Java**Other requirements:** Java 1.5 or higher**License:** GNU LGPL version 3 or higher**Any restrictions to use by non-academics:** no restrictions

## Abbreviations

API: Application programming interface; KiSAO: Kinetic Simulation Algorithm Ontology; LSODE: Livermore Solver for Ordinary Differential Equations; MIASE: Minimal Information About a Simulation Experiment; OWL: Web Ontology Language; SED-ML: Simulation Experiment Description Markup Language.

## Competing interests

The authors declare that they have no competing interests.

## Authors’ contributions

NLN introduced the conception and design of libKiSAO and critically revised the manuscript. CL participated in the design of libKiSAO. RA provided some of the requirements and testing of libKiSAO. AZ developed libKiSAO and drafted the manuscript. All authors read and approved the final manuscript.
